# The Impact of Pain on Functionality, Postural Control and Fall Risk in Woman Aged 45 to 64 Years Old

**DOI:** 10.3390/geriatrics7010010

**Published:** 2022-01-01

**Authors:** Priscilla Beaupré, Rubens A. da Silva, Tommy Chevrette

**Affiliations:** 1 Masters of Biomedical Science Program, Université du Québec à Chicoutimi (UQAC), Saguenay, QC G7H 2B1, Canada; priscilla.beaupre1@uqac.ca (P.B.); rubens_dasilva@uqac.ca (R.A.d.S.); 2 Physical Therapy McGill Program in Extension, Université du Québec à Chicoutimi (UQAC), Saguenay, QC G7H 2B1, Canada; 3 Centre Intersectoriel en Santé Durable, Département des Sciences de la Santé, Université du Québec à Chicoutimi (UQAC), Saguenay, QC G7H 2B1, Canada; 4 Centre Intégré de Santé et Services Sociaux du Saguenay—Lac-Saint-Jean (CIUSSS SLSJ), Specialized Geriatrics Services–La Baie Hospital, Saguenay, QC G7H 7K9, Canada; 5 BioNR Research Lab, Département des Sciences de la santé, Université du Québec à Chicoutimi (UQAC), Chicoutimi, QC G7H 2B1, Canada; 6 Département des Sciences de la santé, Clinique Universitaire de Kinésiologie, Université du Québec à Chicoutimi (UQAC), Chicoutimi, QC G7H 2B1, Canada

**Keywords:** pain, woman, hip, osteoarthritis, aging

## Abstract

Background: Ageing in women is associated with chronic degenerative pain leading to a functional decrease and therefore increase fall risk. It is therefore essential to detect early functional decreases in the presence of pain related to osteoarthritis. Objective: This cross-sectional study aimed to assess the impact of pain on functionality, postural control and fall risk in women aged between 45 to 64 years old. Methods: Twenty-one (21) women aged 45 to 64 were evaluated by clinical and functional measures such as a pain questionnaire (Lequesne Index), functional tests (Stair Step Test, 5 times sit-to-stand, 6MWD, Timed-up and Go) and postural performance (under force platform). Women were classified into 2 groups from the Lequesne Pain Index (PI): low pain (score ≤ 9) and strong pain (score ≥ 10) for subsequent comparisons on functionality (physical and postural control performance). Results: A significant impact was observed between the pain index (strong PI) and 3 of the 4 functional tests carried out including Stair Step Test (*p* = 0.001; *g* = 1.44), walking distance (*p* = 0.003; *g* = 1.31) and Timed-up and Go (*p* = 0.04; *g* = −0.93). The group with a strong PI score reported further poor postural control under force platform compared to the weak pain group. Conclusion: Pain and severity based on the PI index negatively modulate physical and postural control performance in women aged 45 to 64 years old.

## 1. Introduction

Population ageing is a global phenomenon. According to the United Nations, in 2050, 16% of the worldwide population will be over 65 years old, up from 9% in 2019 [1]. Ageing is associated with an increase in age-related health problems such as osteoarthritis (OA) [2]. This problem is defined as a disease characterized by the degeneration of cartilage and its underlying bone within a joint, as well as bony overgrowth. The joints most affected by OA are the weight-bearing joints of the lower limbs [3] followed by hips joints [4] in 25% of people who develop symptomatic hip OA in their lifetime [5]. Hip OA associated with pain has a significant impact on quality of life as well as an increased fall risk [6]. 

Joint tissue breakdown eventually leads to pain and joint stiffness [7]. The impact of hip OA leads to a heavy burden of disease [8]. Several studies have evaluated disability and function in patients with hip OA, such as lower extremity muscle strength [9,10,11], aerobic capacity [12,13] or postural control [14]. All results lead to a decrease in functional capacity on the individual who presents hip OA when compared to control subjects [8,9,10,11,12,13,14]. 

The impaired physical function caused by these symptoms increases fall risk in people with OA. Several studies have shown a strong association between OA and falls [15], especially among women [16,17]. Smith et al. [15] have demonstrated that people who are newly diagnosed with hip OA have a 50% higher risk of experiencing a fall and an 85% higher risk of experiencing a fracture than people of similar age and characteristics, without hip OA. The most common causes of the increased fall risk in people with hip OA are decreased strength and proprioception in the hip joint, leading to greater instability and reduced ability to compensate postural control when made unstable, and curtailing of physical activity [18,19,20,21].

Age is a major factor contributing to the appearance and progression of hip OA [22,23]; it is, however, not the only risk factor. Indeed, female gender [23,24,25,26] and obesity [27,28] are other factors that can contribute to early hip OA. Although it is mostly older people who suffer from hip OA [23], hip complaints can appear at an earlier age [5]. To investigate the impact of hip pain, we constructed a battery of 12 functional tests and postural control measures under force platform in the same experimental design for aging women. The purpose of this study was to assess the physical and functional abilities of women aged 45–64 years old with different levels of hip joint pain.

## 2. Materials and Methods

### 2.1. Study Design

A cross-sectional descriptive study was performed between October 2018 and June 2019 at the *Université du Québec à Chicoutimi* (Quebec, QC, Canada) in the *Clinique universitaire de kinésiologie* and BioNR Laboratory. We used the Quality Assessment Tool for observational cohort and cross-sectional studies [29] to provide quality and consistency.

### 2.2. Participants

A total of 21 women participated voluntarily in this study by convenience. The sample was recruited using a newspaper ad in the *Féderation de l’âge d’Or du Québec*, and UQAC social networks and through a kiosk at the *Salon de la Femme* in Saguenay and selected by one author (PB). Women who fulfilled the inclusion criteria were informed about the study and invited to participate. The inclusion criteria were: (1) being a woman; (2) of caucasian origin and (3) aged between 45 and 64 years old (inclusive). Participants were excluded if they: (1) suffered from a cardiovascular, pulmonary or musculoskeletal disorder that could interfere with the safe performance of physical evaluation, (2) hip arthroplasty or (3) had a self-reported diagnosis of knee osteoarthritis. 

Before testing, all women were classified into two groups according to the Lequesne [30] index (PI), which is a questionnaire validated for evaluating the severity index of hip osteoarthritis, with a total of 10 points (see Supplementary Materials, Table S1: Index of Severity for Osteoarthritis of the Hip by Lequesne et al.). A high score refers to significant impairment, while a lower score refers to modest impairment. The groups were then classified in (1) Low PI (score varying of 0 to 9 = less severe pain) and (2) Strong PI (score ≥ 10 = strong pain).

### 2.3. Data Collection

One two-hour laboratory session was required for all participants. Sociodemographic and anthropometric data were first collected. Then, five tests were conducted: (1) Five Times Sit-to-Stand Test [31]; (2) the Stair Step Test [32]; (3) the Six-Minute Walk Distance [33]; (4) the Timed-up and Go [34] and (5) postural control with a force platform [35]. All tests and measures were performed according to standard protocols. One author (PB) conducted the assessment of the participants with the help of a trained research assistant.

#### 2.3.1. Anthropometric Variables

##### Body Mass Index

The body mass index (BMI) was calculated to assess body fat in the clinical setting. This measurement provides a more accurate measure of total fat mass than body weight assessment alone [36]. The calculation was performed according to the National Institute of Health protocol [37] by dividing weight in kilograms by height in square meters (kg/m^2^).

##### Waist Circumference

Waist circumference measurement is another accurate method of assessing the level of health risk associated with obesity or overweight [38]. Waist circumference measurement was taken according to the recommendations of the National Institutes of Health [37].

#### 2.3.2. Physical and Functional Capacity

##### Five Times Sit-to-Stand Test

The five Times Sit-to Stand Test (FTSST) is reliable for a functional evaluation of lower limb strength. The test was performed according to the standardization proposed by Whitney and Wrisley [31]. It requires the participant to stand up and sit down from a chair with arms crossed over the chest five times, as quickly as possible. The score was the time required to complete five sit-to-stand routines. Two trials are permitted with two minutes of rest between trials. We kept the best time of the two trials.

##### Stair Step Test

The Stair Step Test measures the participant’s ability to alternatively lift each foot and touch the top of a single stair for 20 s. The score was the number of touches executed during the test. Two trials are permitted with a two-minute rest between trials. We kept the best score of the two trials.

##### Six-Minute Walk Distance

The Six-Minute Walk Distance Test (6MWD) was used to assess functional exercise capacity. The participant is encouraged to walk the furthest possible distance in six minutes. The reference values used were those recommended by Enright and Sherill [33]. 

##### Timed-Up and Go

The Timed-up and Go (TUG) was used to measure basic mobility and to assess fall risk. The test was performed according to the standardization proposed by Shumway-Cook and Brauer [34]. The participant was asked to walk a three-meter distance, turn around and return to sit back on a chair. Two trials were planned for the test. The best time was kept for analysis.

##### Postural Control Test on the Force Platform

**Figure 1:**
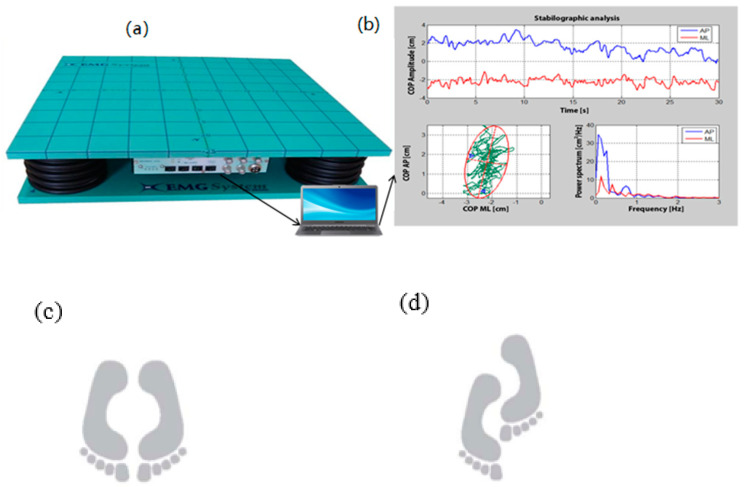
Force plateform and COP measurement. Instrument for measuring with a force platform (BIOMEC400, EMG System do Brasil, Ltda.) (**a**); and postural control measurements based Pressure Center parameter: COP (elliptical area of the COP, frequency and velocity in the anteroposterior and mediolateral directions (**b**). Postural control tasks with a standardized protocol in bipodal (**c**) and semi-tandem (**d**) postures using leg preference in front.

The postural control test for all participants was performed using a force platform (BIOMEC 400, EMG System do Brasil, Ltda, Sao Jose dos Campos, SP, Brazil) during two experimental conditions (Figure 1: (1) bipodal and (2) semi-tandem; both with eyes open and closed. Each condition was performed by two trials of 30 s, with a 1-min rest period between each trial and 2 min between each condition [39]. The mean across 2 trials was retained for subsequent analysis. Overall, all participants were asked to take the requested position standing upright on the platform barefoot, arms at their sides, and with eyes open. They were asked to fix an eye-level target on a wall 2 m away. As a safety measure, a belt was fastened around their waist and a collaborator stood near the participants during the tests to prevent falls.

##### COP Data Processing

The COP-based postural parameters were computed with the use of a validated BIOMEC 400 force platform containing 4 strain gauges arrayed in a rectangle. The sensitivity of each sensor is certified to be 0.0015% for a maximum load of 1000 N and the variation of 9.999 N of the force applied to one strain gauge corresponds to a 120-mV variation of the output. The output range runs from 0 to 5 V. Reaction force signals were sampled at 100 Hz and filtered with a 35-Hz low-pass second-order Butterworth filter and converted into COP data using EMG system do Brasil software which was compiled with MATLAB routines (The Mathworks, Natick, MA, USA). Stabilographic analysis of COP data led to determine the following postural parameters during a 30-s trial: 95% confidence ellipse area of COP (named A-COP: the total area covered in the sagittal and frontal planes using an ellipse, cm^2^) and COP mean Velocity (named VEL: the sum of the cumulated COP displacement divided by the total time, in cm^2^) in the anteroposterior (AP) and mediolateral (ML) directions of movement [40,41,42]. These parameters have been widely used to reflect postural stability, efficiency of the postural control systems and postural performance, respectively, in a variety of contexts [40,41,42].

### 2.4. Data Analysis

The main variables were described by means and standard deviation. The Shapiro–Wilk test was first used to evaluate the normality of the variables and determine which tests would be used. Student’s *t*-test was used to compare the two group of pain (low vs. strong pain). For functional and postural control variables, the two groups were compared by independent Student *t*-test. To determine the magnitude of the differences between two pain groups, the percentage of clinical differences as well as the effect size were calculated according to Glass and Hopkins [43] using Hedge’s coefficient *g* [44] as: small (*g* = 0.20–0.49), moderate (*g* = 0.50–0.79) and large (*g* ≥ 0.80) based on our size sample. Finally, Pearson correlations between clinical measures and physical functional tests and postural control were performed as small (0.1–0.30), moderate (0.30–0.50) and strong (>0.50) [45]. All statistical analyses were performed with a significant alpha risk of less than 0.05. IBM SPSS version 26.0 software (IBM, Armonk, NY, USA) was used for the statistical analysis.

## 3. Results

Sociodemographic (age, occupation, education level, household income, matrimonial status) and anthropometric (BMI and waist circumference) data are presented in Table 1. Overall, both groups (low and strong PI) were homogeneous.

### 3.1. Physical and Functional Capacity and Balance Performance

The pain significantly (*p* < 0.05) affected three physical variables with Hedge’s *g* changes varying from −0.36 to 1.44 across clinical values of performance (Table 2). Women in the strong PI group reported an increase in the time taken to perform the FTSST (+60.57%; *p* ≤ 0.08), a decrease in the number of repetitions of the Stair-Step Test (−40.44%; *p* ≤ 0.001), a decrease in the distance during the Six-minute Walk Distance Test (6MWD: −22.09%; *p* ≤ 0.003) and an increase in the time to perform the TUG (+24.33%; *p* ≤ 0.04) when compared to the low PI group.

Furthermore, significant differences between groups were reported for the amplitude of COP variable (AP) in anterior-posterior direction for the bipodal condition, during eyes open (+20.29%; *p* ≤ 0.03) measures. These results on postural control were not confounded by BMI differences between groups (*p* > 0.05). BMI did not mediate any influence on postural control, suggesting that strong pain would mean poor postural control.

### 3.2. Clinical and Functional and Balance Relationship 

As presented in Table 2, significant correlations were found between pain and FTSST (r ≥ 0.53; *p* ≤ 0.02); Stair-Step Test (r ≥ −0.72; *p* ≤ 0.001); 6MWD in meters (r ≥ −0.64; *p* ≤ 0.003); and TUG (r ≥ 0.50; *p* ≤ 0.03). For the postural control tests, a significant correlation was found between pain and VEL-COP during the bipodal anteroposterior measure with eyes open (r ≥ 0.49; *p* ≤ 0.03). Overall, these correlations varied from weak to moderate across COP variables (r −0.01 to −0.068).

## 4. Discussion

The main finding of our study is that women who report higher hip pain have lower physical and functional capacity. Moreover, higher self-reported hip pain has a negative effect on functional postural control in women aged 45 to 64 years old. Overall, the functional impact of pain was rather negative for the women in this study. To our knowledge, few studies have focused exclusively on the same experimental design of the impact of hip pain on physical and functional capacity and postural control using COP parameters, including a total of 12 variables in the present study (Table 2). 

The significant decrease in functionality related to pain is consistent with previous studies [11,46,47,48,49] and confirms that hip pain is extremely prevalent in an older community-based population. However, some studies have reported a lower significant association between pain and some physical variables [11,14,47]. This difference could be attributed to the smaller sample size in our study or to variability in the choice of tests performed, mainly when comparing groups on postural control variables (only one significant difference was reported for COP values). Our study was also not based on diagnostic criteria for hip OA, but on the subjective perception of hip pain. Overall, the present findings show an association between the intensity of hip pain and several factors that impact physical functions, such as functional variables associated with lower limb strength, speed and walking ability. Although discreet, significant correlations were also pointed out for postural control performance from COP values. High BMI was observed in both groups (mean 27.78 to 32.95), but without affecting postural control results between groups. As reported in Jeanmaire et al. [28], patients with low lean mass showed more pain. They report that in patients with knee and/or hip OA, normal BMI is associated with greater pain and impaired function than in those with higher BMI. Pan et al. [46] found that BMI may be associated with an increased risk of pain at several sites, both at weight-bearing sites such as the lower limbs and at non-weight-bearing sites such as the hands. This finding suggests that body weight has a substantial effect on the pathogenesis of pain. Indeed, it is recognized that adipose tissue serves as an endocrine organ producing cytokines and pro-inflammatory adipokines [50]. As reported by Hartvigsen et al. [51] and by Neogi [52], inflammation can lead to hypersensitivity to pain. It is possible that subjects with a higher BMI are more vulnerable to pain sensitivity. Perhaps the women in this study reported even, low pain experiences because of their body weight.

Moreover, the muscle strength decrease could be explained by the appearance of some form of kinesiophobia and motionlessness caused by pain [28]. Thus, pain could be responsible for a decrease in the amount of physical activity practiced, which could lead to a significant decrease in muscle mass and consequently, in muscle strength [11,28]. Indeed, literature is clear about the fact that muscle weakness and atrophy are characteristics that are present in subjects with osteoarthritis, regardless of the severity of their condition [53,54]. Our study shows a 40% increase in time to perform FTSST in participants in the higher hip pain group. This result is consistent with a previous study conducted by Jerez-Mayorga et al. [49] in which healthy subjects are compared with subjects with osteoarthritis. The performance of subjects with osteoarthritis was 34% slower in the Sit to Stand Test. 

Some of the results of the present study corroborate the findings by Hall et al. [11] which show that greater strength in the hip and thigh muscles may be associated with better self-reported physical function. As reported by Mosler et al. [48] hip pain sufferers demonstrate impaired performance on functional tasks and postural control. Consistent with our study, Mosler, Kemp [48] also reported that hip-related pain demonstrates lower hip strength. The author recommends measuring strength using objective methods of measurement like dynamometry, preferring dynamometers with external fixation to minimize the potential systematic error of a handheld dynamometer, which was not done in our study. 

Muscular strength and power are factors that enable motor and postural control. Decreasing these functions can lead to increased fall risk [55]. Falls are one of the main causes of morbidity and mortality in the adult population [6]. Some studies have shown that hip OA is associated with an increased fall risk [6,14,15,17,18]. De Zwart et al. [56] report that muscular strength is the most significant independent variable associated with fall risk in people with OA. Poor postural control, lower limb muscle weakness, decreased proprioception and pain may contribute to the increased incidence of falls in older adults [14,18]. In contrast to the results obtained in our study, a systematic review of Picorelli [14] reports that no significant difference could be observed in the time taken to perform the TUG. Our results showed that women reporting more pain had a 24% longer duration than women in the low pain group.

Another study conducted by Rydevik et al. [47] compared a functional control group with patients with hip OA. In contrast to our study, they reported no significant differences between the two groups in terms of muscle strength and aerobic capacity. However, as in our study, a significantly shorter distance was walked in the Six-Minute Walk Test. 

The explanation for a lack of association between postural control and pain is not entirely clear. Differences when performing more difficult tasks such as the semi-tandem position may be less important. It is also possible that the range of postural control tests, equipment and duration of tests may have contributed to differences in findings in several studies. As opposed to the systematic review conducted by Picorelli et al. [14], our study has revealed that AP postural control was more impaired than ML postural control. The author showed some inconsistencies. The hip OA group showed greater ML sway with eyes open and greater AP sway with eyes closed compared to controls. Our study showed a significant increase in sway AP with eyes open in the strong PI group. Nevertheless, a trend towards decreased postural control was observed in the tasks performed in our study. This trend for deficits in postural control could be due to neuromuscular contributions.

To our knowledge, our study is the first to evaluate lower limb velocity in women under 65 years old and hip pain without arthroplasty. Our results showed that participants suffering from hip pain took 40% longer to complete the Stair-Step Test than those reporting less pain. These results could be explained by a decrease in muscle strength [11,48].

In addition to the studies of Mosler, Kemp [48] and Damen, van Rijn [57], our study is one of few that presents data on hip pain in younger subjects. Hip OA is a condition that is particularly present in older patients. Early detection of symptoms is essential to implement actions that will allow to maintain optimal physical function and reduce fall risk with ageing.

Therefore, we suggest that future studies validate whether patients with hip pain or hip OA are aged between 45 and 64 years old. It is essential to determine early factors leading to severe hip OA to increase functional capacity, to delay or avoid hip replacement, to reduce fall risk, fractures and eventually morbidity and mortality. An earlier diagnosis would lead to timely interventions to ensure joint health for as long as possible.

### Limitation

Limitations of our study include the use of a self-report questionnaire for pain assessment that did not include frequency and severity of pain. Additionally, the assessment of muscle strength was performed only with the Sit-to-Stand Test. As recommended by Mosler [48], more precise muscle strength measurements, such as those obtained with a dynamometer, would have provided more information on the strength of the lower limbs in several ranges of motion. Another limitation is the very small number of recruited participants. A very small sample size generates relatively low statistical power (possible type-2 errors mainly for postural control COP parameters). Therefore, further studies are required to determine whether hip pain, in this case secondary to hip OA, has a significant impact on anthropometric, physical and functional capacity and postural control in women aged 45–64 years old.

## 5. Conclusions

We observed hip pain, and some physical and functional limitations in our strong pain group. The main results of this study showed that hip pain had a significantly negative impact in the women evaluated on several physical and functional characteristics such as strength, endurance and speed of the lower limbs as well as, to a lower degree, on postural balance. These findings imply a decrease in quality of life in the medium and long term in these women. Further studies are needed to validate whether women 45–64 years old with hip pain could maintain their functional abilities before a diagnosis of OA.

## Figures and Tables

**Table 1:** Sociodemographic and anthropometric outcomes.

Outcomes	Low-PI (*n* = 14)	Strong-PI (*n* = 7)	*p*-Value
Age (years)	56.6 ± 4.7	57.6 ± 5.3	0.66
BMI (kg/m^2^)	27.4 ± 4.4	34.4 ± 9.4	0.11
Waist circumference (cm)	98.0 ± 9.9	108.1 ± 13.5	0.39
Occupation			
Remunerated job	8 (57.1%)	3 (49.2%)	0.44
Retired	5 (35.7%)	2 (28.6%)	
Self-employed	0 (0.0%)	1 (14.3%)	
Housewife	1 (7.1%)	0 (0.0%)	
Disable	0 (0.0%)	1 (14.3%)	
Education level			
Secondary	1 (7.1%)	2 (28.6%)	0.57
Professional	4 (28.6%)	0 (0.0)	
College	2 (14.3%)	1 (14.3%)	
Bachelor	5 (35.7%)	3 (42.9%)	
Graduate and more	2 (14.3%)	1 (14.3%)	
Household income (CAN$)			
<15,000$	0 (0.0%)	1 (14.3%)	0.06
15,000 to 34,999$	3 (30.0%)	0 (0.0%)	
35,000 to 79,999$	1 (10.0%)	4 (57.1%)	
≥80,000$	6 (60.0%)	2 (28.6%)	
Matrimonial status			
Single	1 (7.1%)	2 (28.6%)	0.70
Married	8 (57.1%)	4 (57.1%)	
Common-law partner	4 (28.6%)	1 (14.3%)	
Divorced	1 (7.1%)	0 (0.0%)	

Age, BMI and waist circumference are expressed as mean ± SD (standard derivation). Other Data are expressed as *n* (%). PI = pain index; BMI = body mass index.

**Table 2:** Differences between pain groups on anthropometric, physical capacity and postural control and coefficient correlations across these variables.

Variable	Low-PI *n* = 14 Mean ± SD	Strong-PI *n* = 7 Mean ± SD	Differences (%)	Hedge’s *g*	*p* Value	Pearson’s r (*p* Value)
**Anthropometric**						
BMI (kg/m^2^)	27.78 ± 7.36	32.95 ± 9.58	+18.61	−0.57	0.11	0.36 (0.13)
Waist circumference (cm)	99.59 ± 8.34	104.34 ± 16.43	+4.77	−0.36	0.39	0.17 (0.49)
**Physical and functional capacity**						
FTSST (seconds)	9.73 ± 2.38	13.64 ± 5.17	+40.18	−0.97	0.08	0.53 * (0.02)
Stair Step Test (repetitions)	27.92 ± 5.01	19.88 ± 5.26	−40.44	1.44	0.001 **	−0.72 ** (0.001)
6MWD (meters)	631.82 ± 74.55	517.50 ± 78.61	−22.09	1.31	0.003 **	−0.64 ** (0.003)
TUG (seconds)	5.63 ± 0.95	7.00 ± 1.83	+24.33	−0.93	0.04 *	0.50 * (0.03)
	**Low-PI** ***n* = 12**	**Strong-PI** ***n* = 7**				
**Postural control (force platform)**						
VEL-COP Bipodal AP EO (cm/s)	0.69 ± 0.11	0.83 ± 0.17	+20.29	−0.94	0.03 *	0.49 * (0.03)
VEL-COP Bipodal ML EO (cm/s)	0.72 ± 0.09	0.78 ± 0.18	+8.33	−0.50	0.37	0.22 (0.37)
VEL-COP Bipodal AP EC (cm/s)	0.85 ± 0.15	0.99 ± 0.21	+16.47	−0.73	0.11	0.38 (0.11)
VEL-COP Bipodal ML EC (cm/s)	0.77 ± 0.09	0.80 ± 0.16	+3.90	−0.23	0.68	0.10 (0.68)
VEL-COP Semi-tandem AP EO (cm/s)	1.00 ± 0.17	1.07 ± 0.24	+7.00	−0.32	0.46	0.18 (0.46)
VEL-COP Semi-tandem ML EO (cm/s)	1.34 ± 0.26	1.43 ± 0.32	+6.72	−0.29	0.49	0.17 (0.49)
VEL-COP Semi-tandem AP EC (cm/s)	1.46 ± 0.37	1.58 ± 0.38	+8.22	−0.29	0.51	0.16 (0.51)
VEL-COP Semi-tandem ML EC (cm/s)	2.08 ± 0.60	2.29 ± 0.74	+10.10	−0.29	0.51	0.16 (0.51)

Data are expressed as mean ± SD (standard derivation). PI = pain index; Low-PI = Low Pain Index group (PI ≤ 9) and Strong-PI = higher pain group (PI ≥ 10). BMI = Body Mass Index; FTSST = Five times sit to stand test; 6MWD = 6 min walk distance; TUG = Timed-up and Go; AP = anteroposterior; Postural control: VEL-COP (mean sway velocity of Centre of pressure: COP) parameter. AP = anteroposterior; ML = mediolateral; EO = eyes open; EC = eyes close. * Significant group differences = *p* < 0.05, (Student *t*-test). ** Significant group differences = *p* < 0.01, (Student *t*-test). Pearson’s coefficient correlations between pain measure and anthropometric, physical capacity and postural control variables. Significant correlations between these variables are illustrated for * *p* < 0.05.

## Data Availability

Not applicable.

## Supplementary Materials

The following are available online at https://www.mdpi.com/article/10.3390/geriatrics7010010/s1, Table S1: Index of Severity for Osteoarthritis of the Hip by Lequesne et al.
