# Supplementary material for: The Impact of Pain on Functionality, Postural Control and Fall Risk in Woman Aged 45 to 64 Years Old
Source: Geriatrics (Basel). 2022 Jan 1;7(1):10. doi: 10.3390/geriatrics7010010 (PMC8788291; doi:10.3390/geriatrics7010010)
Supplement: Supplementary file 1 [file geriatrics-07-00010-s001.zip › geriatrics-1532741-supplementary.pdf]

Index of Severity for Osteoarthritis of the Hip by Lequesne et al.

Overview:

Lequesne et al developed an index of severity for osteoarthritis for the hip (ISH). This can be used to assess the effectiveness of therapeutic interventions.

Sections for index:

(1) pain or discomfort

(2) maximum distance walked

(3) activities of daily living

I Pain or Discomfort

**Parameter**	**Finding**	**Points**
pain or discomfort during nocturnal bedrest	none	0
	only on movement or in certain positions	1
	without movement	2
duration of morning stiffness or pain after getting up	none	0
	< 15 minutes	1
	>= 15 minutes	2
remaining standing for 30 minutes increases pain	no	0
	yes	1
pain on walking	none	0
	only after walking some distance	1
	early after starting	2
pain or discomfort in sitting position for 2 hours	no	0
	yes	1

where:

- A modification of a 1991 version was to have the duration of morning stiffness scored 0 if it was 1 minute or less and 1 if it was from 1 to less than 15 minutes.

- Pain on walking in a 1991 version expanded "early after starting" to "after initial ambulation and increasingly with continued ambulation"

II. Maximum Distance Walked

**Parameter**	**Finding**	**Points**
maximum distance walked	unlimited	0
	> 1 kilometer but limited	1
	about 1 kilometer (about 15 minutes)	2
	about 500 - 900 meters (about 8-15 minutes)	3
	from 300 - 500 meters	4
	from 100 - 300 meters	5
	< 100 meters	6
walking aids required	none	0
	1 walking stick or crutch	1
	2 walking sticks or crutches	2

III. Activities of Daily Living

**Parameter**	**Finding**	**Points**
Can you put on socks by bending forward?	easily	0
	with mild difficulty	0.5
	with moderate difficulty	1.0
	with marked difficulty	1.5
	impossible	2.0
Can you pick up an object from the floor?	easily	0
	with mild difficulty	0.5
	with moderate difficulty	1.0
	with marked difficulty	1.5
	impossible	2.0
Can you go up and down a standard flight of stairs?	easily	0
	with mild difficulty	0.5
	with moderate difficulty	1.0
	with marked difficulty	1.5
	impossible	2.0
Can you get into and out of a car?	easily	0

	with mild difficulty	0.5
	with moderate difficulty	1.0
	with marked difficulty	1.5
	impossible	2.0

index of severity =

= SUM(points for all parameters)

Interpretation:

• minimum points for each section: 0

• maximum points for each section: 8

• minimum index score: 0

• maximum index score: 24

Index Score	Handicap
0	none
1 - 4	mild
5 - 7	moderate
8 - 10	severe
11 - 13	very severe
>= 14	extremely severe

Modifications to Index

The index was modified in 1991 (Table 2) by the adddition of a question for sexual activity in sexually active women being evaluated for hip prosthesis. This was graded as for the activities of daily living. This results in a maximum index score of 26.

The index was modified in 1997 with some minor changes to morning stiffnes and termed the "algofunctional index".

References:

Lequesne M Mery C et al. Indexes of severity for osteoarthritis of the hip and knee. Scand J Rheumatology. 1987; Supplement 65: 85-89.

Lequesne M. Indices of severity and disease activity for osteoarthritis. Seminars in Arthritis and Rheumatism. 1991; 20 (supplement 2): 48-54.

Lequesne MG. The algofunctional indices for hip and knee osteoarthritis. J Rheumatol. 1997; 24: 779-781.
